# Supplementary material for: Psychosocial factors associated with mental health and quality of life during the COVID-19 pandemic among low-income urban dwellers in Peninsular Malaysia
Source: PLoS One. 2022 Aug 23;17(8):e0264886. doi: 10.1371/journal.pone.0264886 (PMC9398022; doi:10.1371/journal.pone.0264886)
Supplement: S1 Table — (PDF) [file pone.0264886.s001.pdf]

S1 Table for sample size according to study objectives

Objectives	Literature	Proportion/OR /Mean Difference	Inflated (10% Reserved Rate) Sample Size
Prevalence			
**Depressive and Anxiety symptoms**	Sareen, Afifi et al. 2011<br><br>Tan et al. 2012	Prevalence of depression among low-income Depression:27.1%, GAD: 10.3%<br>Prevalence of Depression: 12.1%	332<br><br>410
**Quality of Life (WHQOL-Bref)**	Cao et al 2016	Association of the mental disorders and quality of life outcome. The proportion of poor QOL from the Psychological domain is 11.6%	348
Associated Factors			
**Gender**	Tan and Yadav 2019<br>Cheah et al. 2020	Depression: Male > Female<br>Depression :Female >Male : OR =2.9	517<br>450
**Employment status**	McGee and Thompson 2015	OR =3.25	136
**Smoking /substance used UK Urban poor and Depression**	Adult Psychiatric Morbidity Survey(NHS 2014)	Exposed and non-exposed with the outcome Depression:2.4 Vs 9.9 GAD: 4.6 Vs 13.8	444
**Health Literacy**	Lopez, Sanchez et al. 2018	Mean Difference for low health literacy and high literacy	233
**Attribution of Poverty/low socio-economic**	Eaton, Muntaner et al. 2001	OR :3.3	396

Information Used to Calculate the Sample Size

$$n = Z^2 p (1-p) / e^2$$

Variables	*Definition / value*
z	*the standard normal deviate, usually set at 1.96 at 5% level for a two-tailed test which corresponds to a 95% confidence level*
p	*estimated prevalence*
n	*the desired sample size which would be sufficient to measure the different variables*
e	*the precision level or the distance from the prevalence estimate in either direction. It has been set to ± 5% point for two tails equation or 0.05 (expressed in decimal)*
