# Supplementary material for: Psychosocial factors associated with mental health and quality of life during the COVID-19 pandemic among low-income urban dwellers in Peninsular Malaysia
Source: PLoS One. 2022 Aug 23;17(8):e0264886. doi: 10.1371/journal.pone.0264886 (PMC9398022; doi:10.1371/journal.pone.0264886)
Supplement: S2 Table — (PDF) [file pone.0264886.s002.pdf]

S2 Table for Cronbach's alpha and correlation coefficients for tools used in the pilot study

Tools	Cronbach alpha Coefficient	Test-Retest Reliability
**EQ-5D-5L**		
EQ-Index		*0.86
EQ-VAS		*0.98
**Descriptive System**		
Mobility		**0.55
Self-care		**0.65
Usual activities		**0.64
Pain		**0.48
Anxiety or depression.		**0.72
**PHQ-9**	0.74	*0.92
**GAD-7**	0.89	*0.98
**Health Literacy HLS-SF6**	0.90	*0.84
**Poverty Attribution- 21**	0.93	*0.98
Structural	0.86	*0.98
Structural Economy	0.87	*0.98
Individualistic	0.93	*0.98
Fatalistic	0.83	*0.96
**Mental Health Help Seeking Attitude Scale (MHSAS)**	0.89	*0.92
**Resilience-14**	0.96	*0.95
**Self-Stigma of Seeking Help Scale (SSOSHS)**	0.84	*0.98
**Religiosity (SCSORF)**	0.79	*0.94

*pearson correlation p< 0.001 ** Kappa coefficient
