# Supplementary material for: Psychosocial factors associated with mental health and quality of life during the COVID-19 pandemic among low-income urban dwellers in Peninsular Malaysia
Source: PLoS One. 2022 Aug 23;17(8):e0264886. doi: 10.1371/journal.pone.0264886 (PMC9398022; doi:10.1371/journal.pone.0264886)
Supplement: S3 Table — (PDF) [file pone.0264886.s003.pdf]

S3 Table for health-related profiles of b40 respondents from the Petaling district

Variables	Total	n (%)	Mean (SD) / Median (IQR)
Weight (Kg)	430		70.0 (15.3)[2]
Height (m)	430		1.7 (0.1)[2]
BMI (kg/m^2)	430		25.0 (5.7)[2]
**BMI categories**			
<18.5 kg/m2	430	29 (6.9)	
18.5-22.9 kg/m2		97 (22.9)	
23.0-27.4 kg/m2		172 (40.6)	
27.5- 34.9 kg/m2		107 (25.3)	
≥ 35.0 kg/m2		18 (4.3)	
**History of chronic illness (NCDs)**			
Yes	428	130 (30.4)	
No		298 (69.6)	
**Details of NCDs**	428		
Hypertension		78 (18.2)	
Diabetes Mellitus		38 (8.9)	
Heart disease		15 (3.5)	
Kidney disease		7 (1.6)	
Cancer		3 (0.7)	
Exhaustion		20 (4.7)	
Stroke		1 (0.2)	
Mental illness		2 (0.5)	
Others		22 (5.1)	
**Presence of stressful events**			
Yes	431	209 (48.5)	
No		222 (51.5)	
**Details of stressful events**			
Assault (physical and sexual)		25 (5.8)	
Prolonged serious illnesses		32 (7.4)	
Bullied during childhood		26 (6.0)	
Injury due to accident		34 (7.9)	
Orphan below 10 years old		11 (2.6)	
Loss of loved one		150 (34.8)	
Marital issue		22 (5.1)	
Family issue		28 (6.5)	
Financial issue		26 (6.0)	
Neighborhood issue		19 (4.4)	
Working environment issue		48 (11.1)	
Loss of job		48 (11.1)	
Legal issue		17 (3.9)	
**Substances use**			
Yes	431	130 (30.2)	
No		301 (69.8)	
**Details of substance use**			
Smoking	431	116 (26.9)	
Alcohol		33 (7.7)	
Cannabis		3 (0.7)	
Cocaine		1 (0.2)	

Amphetamine-related		1 (0.2)	
Inhaler		2 (0.5)	
Sleeping pill		8 (1.9)	
Hallucinogen		2 (0.5)	
Opioid		1 (0.2)	
Others		4 (0.9)	
**Health Related Quality of Life (EQ-5D-5L)**			
**Descriptive system**			
With problem (< 1.0)	431	119 (27.6)	
Without problem (=1.0)		312 (72.4)	
**Descriptive System with Breakdown**	428		
**Mobility**			
No problem		377(88.1)	
Problem		51(11.9)	
**Self-care**			
No problem		414 (96.7)	
Problem		14(3.3)	
**Usual Activities**			
No Problem		380(89.0)	
Problem		47(11.0)	
**Pain/ Discomfort**			
No Problem		332(77.6)	
Problem		96(22.4)	
**Depression / Anxiety**			
No Problem		361(84.4)	
Problem		67(15.7)	
**Value set (EQ-index)**			1.0 (0.8)[2]
**Scale (ED-VAS)**	429		85(15)[2]
Bad (<65)		53 (12.4)	
Fair (65-79)		48 (11.2)	
Good (80-89)		137 (31.9)	
Excellent (>90)		191 (44.5)	
**Mental health status**			
**PHQ-9 total score**	429		3.0 (4.0)[2]
**GAD-7 total score**	430		1.0 (3.0)[2]
**PHQ-9 (Depression-diagnosis)**			
<10	429	399 (93.0)	
≥10		30 (7.0)	
**GAD-7 (Anxiety-diagnosis)**			
<8	430	411 (95.6)	
≥8		19 (4.4)	
**PHQ-9 (Depression- symptomatic)**	429	302 (70.4)	

<5		127 (29.6)	
≥5			
**GAD-7 (Anxiety-symptomatic)**			
<5	430	367 (85.0)	
≥5		63 (14.6)	
**PHQ-9**	429		
Normal (0-4)		302(70.40)	
Mild (5-9)		97(22.61)	
Moderate to Severe (>=10)		30 (6.99)	
**GAD-7**			
Normal (0-4)	429	366(85.31)	
Mild (5-8)		44(10.26)	
Moderate to Severe (>=8)		19(4.43)	

^{1} = Mean (SD); ^{2} = Median (IQR)
