# Supplementary material for: Psychosocial factors associated with mental health and quality of life during the COVID-19 pandemic among low-income urban dwellers in Peninsular Malaysia
Source: PLoS One. 2022 Aug 23;17(8):e0264886. doi: 10.1371/journal.pone.0264886 (PMC9398022; doi:10.1371/journal.pone.0264886)
Supplement: S4 Table — (PDF) [file pone.0264886.s004.pdf]

S4 Table for descriptive output for psychosocial risk factors of the B40 respondents from the Petaling district (n=432)

Variables	Total	n (%)	Mean (SD) / Median (IQR)
**Religiosity**	432		17.0 (5.0)[2]
**Religiosity category**	432		
**Low (< 17)**		182 (42.1)	
**High (≥ 17)**		250 (57.9)	
**Poverty attribution Average**	431		3.4 (0.6)[1]
**Poverty attribution (Structural) Average**	431		3.6 (0.9)[1]
**Poverty attribution (Socioeconomic)Average**	431		3.8 (0.8)[1]
**Poverty attribution (Individualistic)Average**	431		3.4 (1.1)[1]
**Poverty attribution (Fatalistic)Average**	431		3.0 (1.0)[1]
**Health literacy**	428		
Average			3.0 (0.6)[1]
Health literacy index			33.4 (10.8)[1]
**Help seeking (MHSAS)**	429		
Total			54.0 (18.0)[2]
Average			6.0 (2.0)[2]
**Self-stigma (SSOSHS)**	430		
Total			24.9 (5.8)[1]
Average			2.5 (0.6)[1]
**Resilience**	429		
Total			73.2 (14.7)[1]
**Categorical**			
Low		133 (31.0)	
Moderate		162 (37.8)	
High		134 (31.2)	

[1] = Mean (SD); [2] = Median (IQR).
