# Supplementary material for: Psychosocial factors associated with mental health and quality of life during the COVID-19 pandemic among low-income urban dwellers in Peninsular Malaysia
Source: PLoS One. 2022 Aug 23;17(8):e0264886. doi: 10.1371/journal.pone.0264886 (PMC9398022; doi:10.1371/journal.pone.0264886)
Supplement: S5 Table — (PDF) [file pone.0264886.s005.pdf]

Table A. Univariable analysis on socio-demographic factors associated with depression (symptomatic) among B40 respondents from the Petaling district (n=432)

Socio-demographic variables	Depression (symptomatic)		Test statistic	*p* value
	≥ 5	< 5		
**Gender (n=428)**				
Male	77 (27.9)	199 (72.1)	1.172[a]	0.279
Female	50 (32.9)	102 (67.1)		
**Age (n=410)**	40.7 (13.1)	44.2 (13.1)	2.453[b]	0.015
**Age category (n=410)**				
< 30	30 (46.9)	34 (53.1)	11.223[a]	0.011
30-40	37 (28.2)	94 (71.8)		
41-50	30 (28.8)	74 (71.2)		
> 50	26 (23.4)	85 (76.6)		
**Ethnicity (n=427)**				
Malay	88 (27.3)	234 (72.7)	3.205[a]	0.361
Chinese	18 (31.6)	39 (68.4)		
Indian	12 (40.0)	18 (60.0)		
Others	7 (38.9)	11 (61.1)		
**Marital status (n=427)**				
Single/Divorced	27 (36.5)	47 (63.5)	2.298[a]	0.317
Widowed	10 (29.4)	24 (70.6)		
Married	88 (27.6)	231 (72.4)		
**Education level (n=426)**				
No formal Education and - Primary	16(55.2)	13(44.8)	10.59	0.005
Secondary school	53(25.7)	153(74.3)		
>Secondary	57(29.8)	134(70.2)		
**Employment status before outbreak (n=424)**				
Yes	108 (30.4)	247 (69.6)	0.229[a]	0.632
No	19 (27.5)	50 (72.5)		
**Employment status during outbreak (n=411)**				
Yes	17 (6.2)	258 (93.8)	0.203[a]	0.652
No	10 (7.4)	126 (92.6)		
**Household income before outbreak (n=429)**	3000.0 (3200.0)	3000.0 (2200.0)	19119.000[c]	0.961
**Household income before outbreak groups (n=429)**				
<RM 1700	30 (35.7)	54 (64.3)	10.191[a]	0.070
RM 1700 to 2700	29 (29.0)	71 (71.0)		
RM 2701 to 3700	23 (23.7)	74 (76.3)		
RM 3701 to 4700	12 (19.4)	50 (80.6)		
RM 4700-5700	12 (33.3)	24 (66.7)		
>RM 5701	21 (42.0)	29 (58.0)		

**Household income during outbreak (n=429)**	2682.1 (1986.1)	2845.7 (1805.8)	0.831[b]	0.406
**Household income during outbreak groups (n=429)**				
<RM 1700	46 (34.6)	87 (65.4)	3.473[a]	0.628
RM 1700 to 2700	23 (25.3)	68 (74.7)		
RM 2701 to 3700	20 (26.0)	57 (74.0)		
RM 3701 to 4700	15 (27.3)	40 (72.7)		
RM 4700-5700	9 (28.1)	23 (71.9)		
>RM 5701	14 (34.1)	27 (65.9)		
**Household size (n=423)**				
≥ 4	68 (29.3)	164 (70.7)	0.056[a]	0.813
< 4	58 (30.4)	133 (69.6)		
**Years-live in residential area (n=398)**				
≤ 10 years	57 (30.2)	132 (69.8)	0.012[a]	0.914
> 10 years	62 (29.7)	147 (70.3)		
**Years-live in residential area (n=398)**				
≤ 4 years	26 (34.2)	50 (65.8)	0.833[a]	0.361
> 4 years	93 (28.9)	229 (71.1)		
**House-ownership (n=426)**				
Owners/Inherited	55 (28.1)	141 (71.9)	0.401[a]	0.527
Shelter/Renting	71 (30.9)	159 (69.1)		
**Asset- House (n=426)**				
Yes	63 (27.4)	167 (72.6)	1.147[a]	0.284
No	63 (32.1)	133 (67.9)		
**Asset-Vehicle (n=427)**				
Yes	101 (28.9)	248 (71.1)	0.297[a]	0.586
No	25 (32.1)	53 (67.9)		
**Asset-Land (n=425)**				
Yes	14 (34.1)	27 (65.9)	0.440[a]	0.507
No	112 (29.2)	272 (70.8)		
**Asset-Orchard (n=427)**				
Yes	6 (26.1)	17 (73.9)	0.148[a]	0.701
No	120 (29.9)	282 (70.1)		
**Asset-Cash (n=426)**				
Yes	40 (27.0)	108 (73.0)	0.708[a]	0.400
No	86 (30.9)	192 (69.1)		
**Asset-Rental house (n=427)**				
Yes	21 (38.2)	34 (61.8)	2.206[a]	0.137
No	105 (28.4)	265 (71.6)		
**Asset-Jewelry (n=425)**				
Yes	37 (29.1)	90 (70.9)	0.023[a]	0.880
No	89 (29.9)	209 (70.1)		
**Asset-Shophouse (n=425)**				

Yes	5 (83.3)	1 (16.7)	8.410[d]	0.010
No	121 (28.9)	298 (71.1)		
**Asset-Investment (n=425)**				
Yes	21 (42.9)	28 (57.1)	4.634[a]	0.031
No	105 (27.9)	271 (72.10		
**Asset-Others (n=424)**				
Yes	2 (50.0)	2 (50.0)	0.795[d]	0.586
No	124 (29.5)	296 (70.5)		
**Communication tools**				
**Own mobile or smartphone**				
Yes	99 (29.1)	241(70.9)	[a]0.0710	0.790
No	23(30.7)	52(69.3)		
**Own at least a form of communication tool (TV Or Radio and Internet or laptop and Smartphone/ mobile)**				
Yes	116(28.6)	289(71.4)	[d]4.6235	0.041
No	6 (60.0)	4(40.0)		
**Religiosity (n=429)**	17.0 (5.0)	18.0 (5.0)	15199.000[c]	0.001
**Religiosity category (n=429)**				
Low (<17)	60 (33.3)	120 (66.7)	2.070[a]	0.150
High (≥17)	67 (26.9)	182 (73.1)		
**Community connectedness (n=429)**				
once a week	73 (26.0)	208 (74.0)	5.136[a]	0.023
< once a month	54 (36.5)	94 (63.5)		

[a] = Pearson Chi-square; [b]= Independent t-test; [c]= Mann-Whitney U test; [d] = Fischer's exact test.

Table B. Univariable analysis on health-related profiles associated with with depression (symptomatic) among B40 respondents from the Petaling district (n=432)

**Health-related variables**	**Depression (symptomatic)**		**Test statistic**	***p* value**
	**≥ 5**	**< 5**		
**BMI (kg/m²) (n=427)**	24.2 (6.3)	25.4 (5.5)	17531[c]	0.218
**BMI categories (n=427)**				
<18.5 kg/m²	8 (27.6)	21 (72.4)	7.826[a]	0.098
18.5-22.9 kg/m²	39 (40.6)	57 (59.4)		
23.0-27.4 kg/m²	42 (24.7)	128 (75.3)		
27.5- 34.9 kg/m²	30 (28.0)	77 (72.0)		
≥ 35.0 kg/m²	6 (33.3)	12 (66.7)		
**History of chronic illnesses (n=425)**				
Yes	74 (25.1)	221 (74.9)	7.814[a]	0.005
No	50 (38.5)	80 (61.5)		
**Hypertension (n=425)**				
Yes	29 (37.2)	49 (62.8)	2.961[a]	0.085
No	95 (27.4)	252 (72.6)		
**Diabetes Mellitus (n=425)**				
Yes	11 (28.9)	27 (71.1)	0.001[a]	0.974
No	113 (29.2)	274 (70.8)		
**Heart disease (n=425)**				
Yes	7 (46.7)	8 (53.3)	2.302[d]	0.150
No	117 (28.5)	293 (71.5)		
**Kidney disease (n=425)**				
Yes	5 (71.4)	2 (28.6)	6.149[d]	0.025
No	119 (28.5)	299 (71.5)		
**Cancer (n=425)**				
Yes	1 (33.3)	2 (66.7)	0.025[d]	1.000
**No**	123 (29.1)	299 (70.9)		
**Exhaustion (n=424)**				
Yes	8 (40.0)	12 (60.0)	1.231[a]	0.267
No	115 (28.5)	289 (71.5)		
**Stroke (n=426)**				
Yes	1 (100.0)	0	2.433[d]	0.292
No	123 (29.0)	301 (71.0)		
**Mental illness (n=425)**				
Yes	1 (50.0)	1 (50.0)	0.422[d]	0.499
No	123 (29.1)	300 (70.9)		
**Other chronic illnesses (n=425)**				
Yes	16 (72.7)	6 (27.3)	21.296[a]	< 0.001
No	108 (26.8)	295 (73.2)		
**Stressful life event (n=429)**				
No	42 (20.3)	165 (79.7)	16.651[a]	< 0.001
Yes	85 (38.3)	137 (61.7)		

**Assault (n=429)**				
No	110 (27.2)	294 (72.8)	18.780[a]	< 0.001
**Yes**	17 (68.0)	8 (32.0)		
**Prolonged serious illness (n=429)**				
No	106 (26.7)	291 (73.3)	21.530[a]	< 0.001
Yes	21 (65.6)	11 (34.4)		
**Bullied during childhood(n=429)**				
No	115 (28.5)	288 (71.5)	3.638[a]	0.056
Yes	12 (46.2)	14 (53.8)		
**Injury due to accident (n=429)**				
No	110 (27.8)	285 (72.2)	7.371[a]	0.007
Yes	17 (50.0)	17 (50.0)		
**Orphan below 10 years old (n=429)**				
No	124 (29.7)	294 (70.3)	0.029[d]	1.000
Yes	3 (27.3)	8 (72.7)		
**Loss of loved one (n=429)**				
No	74 (26.5)	205 (73.5)	3.633[a]	0.057
Yes	53 (35.3)	97 (64.7)		
**Marital issue (n=429)**				
No	113 (27.8)	294 (72.2)	12.888[a]	< 0.001
Yes	14 (63.6)	8 (36.4)		
**Family issue (n=429)**				
No	107 (26.7)	294 (73.3)	25.145[a]	< 0.001
Yes	20 (71.4)	8 (28.6)		
**Financial issue (n=429)**				
No	111 (27.5)	292 (72.5)	13.544[a]	< 0.001
Yes	16 (61.5)	10 (38.5)		
**Neighborhood issue (n=429)**				
No	117 (28.5)	293 (71.5)	5.059[a]	0.025
Yes	10 (52.6)	9 (47.4)		
**Working environment issue (n=429)**				
No	101 (26.5)	280 (73.5)	15.647[a]	< 0.001
Yes	26 (54.2)	22 (45.8)		
**Loss of job (n=429)**				
No	100 (26.2)	281 (73.8)	18.414[a]	< 0.001
Yes	27 (56.3)	21 (43.8)		
**Legal issue (n=429)**				
No	117 (28.4)	295 (71.6)	7.252[a]	0.007
Yes	10 (58.8)	7 (41.2)		
**Substance use (n=429)**				

No	78 (26.1)	221 (73.9)	5.856[a]	0.016
Yes	49 (37.7)	81 (62.3)		
**Smoking (n=429)**				
No	82 (26.2)	231 (73.8)	6.442[a]	0.011
Yes	45 (38.8)	71 (61.2)		
**Alcohol (n=429)**				
No	110 (27.8)	286 (72.2)	8.236[a]	0.004
Yes	17 (51.5)	16 (48.5)		
**Cannabis (n=429)**				
No	125 (29.3)	301 (70.7)	1.991[d]	0.210
**Yes**	2 (66.7)	1 (33.3)		
**Cocaine (n=429)**				
No	127 (29.7)	301 (70.3)	0.422[d]	1.000
Yes	0	1 (100.0)		
**Amphetamine-related (n=429)**				
No	127 (29.7)	301 (70.3)	0.422[d]	1.000
Yes	0	1 (100.0)		
**Inhaler (n=429)**				
No	126 (29.5)	301 (70.5)	0.401[d]	0.505
Yes	1 (50.0)	1 (50.0)		
**Sleeping pill (n=429)**				
No	122 (29.0)	299 (71.0)	4.233[d]	0.053
Yes	5 (62.5)	3 (37.5)		
**Hallucinogen (n=429)**				
No	126 (29.5)	301 (70.5)	0.401[d]	0.505
Yes	1 (50.0)	1 (50.0)		
**Opioid (n=428)**				
No	127 (29.7)	300 (70.3)	0.423[d]	1.000
Yes	0	1 (100.0)		
**Others (n=428)**				
No	125 (29.5)	299 (70.5)	0.799[d]	0.585
Yes	2 (50.0)	2 (50.0)		

[a] = Pearson Chi-square; [b]= Independent t-test; [c]= Mann-Whitney U test; [d] = Fischer's exact test.

Table C. Univariable analysis on barriers to help seeking associated with depression among B40 respondents from the Petaling district (n=432)

Barriers to help seeking	Depression (symptomatic)		Test statistic	*p* value
	≥ 5	< 5		
**Overall average poverty attribution (n=429)**	3.43 (0.76)	3.48 (0.90)	17237.000[c]	0.098
**Structural (average) (n=429)**	3.40 (1.00)	3.80 (1.20)	14638.000[c]	< 0.001
**Socioeconomic (average) (n=429)**	3.80 (1.00)	4.00 (1.20)	17843.500[c]	0.253
**Individualistic (average) (n=429)**	3.40 (1.60)	3.60 (1.80)	17049.500[c]	0.068
**Fatalistic (average) (n=429)**	3.17 (1.30)	3.00 (1.20)	17709.000[c]	0.209
**Health literacy (average) (n=426)**	3.00 (0.67)	3.00 (0.93)	14148.500[c]	< 0.001
**Health literacy index (n=426)**	33.33 (11.11)	33.33 (15.56)	14148.500[c]	< 0.001
**Help seeking (total) (n=424)**	51.00 (15.00)	57.00 (15.00)	13720.500[c]	< 0.001
**Help seeking (average) (n=424)**	5.67 (1.67)	6.33 (1.67)	13720.500[c]	< 0.001
**Self-stigma (total) (n=428)**	27.00 (7.00)	26.00 (9.00)	16398.000[c]	0.024
**Self-stigma (average) (n=428)**	2.70 (0.70)	2.60 (0.90)	16398.000[c]	0.024
**Resilience (total) (n=427)**	66.00 (20.00)	75.00 (20.50)	12739.500[c]	< 0.001
**Resilience categorical (n=427)**				
Low	55 (41.7)	77 (58.3)	20.174[a]	< 0.001
Moderate	49 (30.2)	113 (69.8)		
High	22 (16.5)	111 (83.5)		

[a] = Pearson Chi-square; [c]= Mann-Whitney U test

Table D. Univariable analysis on socio-demographic factors associated with anxiety (symptomatic) among B40 respondents from the Petaling district (n=432)

**Socio-demographic variables**	**Anxiety (symptomatic)**		**Test statistic**	***p* value**
	**≥ 5**	**< 5**		
**Gender (n=429)**				
Male	37 (13.4)	240 (86.6)	1.100[a]	0.294
Female	26 (17.1)	126 (82.9)		
**Age (n=411)**	40.5 (13.21)	43.6 (13.13)	1.718[b]	0.087
**Age category (n=411)**				
< 30	14 (21.9)	50	4.030[a]	0.258
30-40	21 (16.0)	110		
41-50	13 (12.5)	91		
> 50	13 (11.6)	99		
**Ethnicity (n=428)**				
Malay	45 (14.0)	277 (86.0)	0.581[a]	0.901
Chinese	10 (17.2)	48 (82.8)		
Indian	5 (16.7)	25 (83.3)		
Others	3 (16.7)	15 (83.3)		
**Marital status (n=428)**				
Single/Divorced	13 (17.6)	61 (82.4)	1.830[a]	0.401
Widowed	7 (20.6)	27 (79.4)		
Married	43 (13.4)	277 (86.6)		
**Education level (n=426)**				
No formal Education / Primary	7(24.1)	22(75.9)	3.970[a]	0.137
Secondary school	24(11.7)	182 (88.4)		
>Secondary	31(16.2)	160 (83.8)		
**Employment status before outbreak (n=425)**				
Yes	55 (15.4)	301 (84.6)	0.680[a]	0.409
No	8 (11.6)	61 (88.4)		
**Employment status during outbreak (n=412)**				
Yes	39 (14.1)	237 (85.9)	0.126[a]	0.723
No	21 (15.4)	115 (84.6)		
**Household income before outbreak (n=430)**	3000.0 (2600.0)	3000.0 (2400.0)	11116.500[c]	0.626
**Household income before outbreak groups (n=430)**				
<RM 1700	13 (15.5)	71 (84.5)	3.224[a]	0.665
RM 1700 to 2700	16 (16.0)	84 (84.0)		
RM 2701 to 3700	13 (13.4)	84 (86.6)		
RM 3701 to 4700	6 (9.7)	56 (90.3)		
RM 4700-5700	8 (22.2)	28 (77.8)		
>RM 5701	7 (13.7)	44 (86.3)		
**Household income during outbreak (n=430)**	2682.2 (1824.3)	2809.4 (1871.2)	0.500[b]	0.617

**Household income during outbreak groups (n=430)**				
<RM 1700	19 (14.2)	115 (85.8)	1.997[a]	0.850
RM 1700 to 2700	14 (15.4)	77 (84.6)		
RM 2701 to 3700	10 (13.0)	67 (87.0)		
RM 3701 to 4700	10 (18.2)	45 (81.8)		
RM 4700-5700	6 (18.8)	26 (81.3)		
>RM 5701	4 (9.8)	37 (90.2)		
**Household size (n=423)**				
< 4	26 (13.6)	165 (86.4)	0.304[a]	0.582
≥ 4	36 (15.5)	196 (84.5)		
**Years-live in residential area (n=398)**				
≤ 10 years	26 (13.8)	163 (86.2)	0.094[a]	0.760
> 10 years	31 (14.8)	178 (85.2)		
**Years-live in residential area (n=398)**				
≤ 4 years	12 (15.8)	64 (84.2)	0.165[a]	0.685
> 4 years	45 (14.0)	277 (86.0)		
**House-ownership (n=427)**				
Owners/Inherited	23 (11.7)	174 (88.3)	2.757[a]	0.097
Shelter/Renting	40 (17.4)	190 (82.6)		
**Asset- House (n=427)**				
Yes	30 (13.0)	201 (87.0)	1.249[a]	0.264
No	33 (16.8)	163 (83.2)		
**Asset-Vehicle (n=428)**				
Yes	46 (13.2)	303 (86.8)	3.568[a]	0.059
No	17 (21.5)	62 (78.5)		
**Asset-Land (n=426)**				
Yes	7 (16.7)	3 (83.3)	0.130[a]	0.718
No	56 (14.6)	328 (85.4)		
**Asset-Orchard (n=426)**				
Yes	4 (17.4)	19 (82.6)	0.131[d]	0.761
No	59 (14.6)	344 (85.4)		
**Asset-Cash (n=427)**				
Yes	19 (12.8)	129 (87.2)	0.661[a]	0.416
No	44 (15.8)	235 (84.2)		
**Asset-Rental house (n=427)**				
Yes	11 (20.0)	44 (80.0)	1.361[a]	0.243
No	52 (14.0)	319 (86.0)		
**Asset-Jewelry (n=426)**				
Yes	16 (12.5)	112 (87.5)	0.761[a]	0.383
No	47 (15.8)	251 (84.2)		
**Asset-Shophouse (n=426)**				
Yes	1 (16.7)	5 (83.3)	0.017[d]	1.000
No	62 (14.8)	358 (85.2)		

**Asset-Investment (n=426)**				
Yes	9 (18.4)	40 (81.6)	0.563[a]	0.453
No	54 (14.3)	323 (85.7)		
**Asset-Others (n=426)**				
Yes	0	4 (100.0)	0.703[d]	1.000
No	63 (15.0)	358 (85.0)		
Communication tools				
**Own mobile or smartphone**				
Yes	50 (14.7)	290 (85.3)	[a]0.0001	0.993
No	11(14.7)	64 (85.3)		
**Own at least a form of communication tool (TV Or Radio and Internet or laptop and Smartphone/ mobile)**				
Yes	60(14.8)	345(85.2)	0.1804	0.554
No	1 (10.0)	9(90.0)		
**Religiosity (n=430)**	16.0 (4.0)	18.0 (5.0)	9043.500[c]	0.005
**Religiosity category (n=430)**				
Low (<17)	32 (17.7)	149 (82.4)	2.292[a]	0.130
High (≥17)	31 (12.4)	218 (87.6)		
**Community connectedness (n=430)**				
once a week	31 (11.0)	251 (89.0)	8.769[a]	0.003
< once a month	32 (21.6)	116 (78.4)		

[a] = Pearson Chi-square; [b]= Independent t-test; [c]= Mann-Whitney U test; [d] = Fischer's exact test.

Table E. Univariable analysis on health-related profiles associated with Anxiety Symptoms among B40 respondents from the Petaling district (n=432)

Health-related variables	Anxiety (symptomatic)		Test statistic	*p* value
	≥ 5	< 5		
**BMI (kg/m²) (n=428)**	23.7 (7.29)	25.3 (5.24)	10479.000[c]	0.336
**BMI categories (n=428)**				
<18.5 kg/m²	4 (13.8)	25 (86.2)	9.911[d]	0.034
18.5-22.9 kg/m²	22 (22.9)	74 (77.1)		
23.0-27.4 kg/m²	16 (9.4)	155 (90.6)		
27.5- 34.9 kg/m²	16 (15.0)	91 (85.0)		
≥ 35.0 kg/m²	4 (22.2)	14 (77.8)		
**History of chronic illnesses (n=426)**				
Yes	36 (12.2)	260 (87.8)	3.679[a]	0.055
No	25 (19.2)	105 (80.8)		
**Hypertension (n=426)**				
Yes	15 (19.2)	63 (80.8)	1.877[a]	0.171
No	46 (13.2)	302 (86.8)		
**Diabetes Mellitus (n=426)**				
Yes	6 (15.8)	32 (84.2)	0.074[a]	0.786
No	55 (14.2)	333 (85.5)		
**Heart disease (n=426)**				
Yes	4 (26.7)	11 (73.3)	1.932[d]	0.248
No	57 (13.9)	354 (86.1)		
**Kidney disease (n=426)**				
Yes	3 (42.9)	4 (57.1)	4.724[d]	0.064
No	58 (13.8)	361 (86.2)		
**Cancer (n=426)**				
Yes	0	3 (100.0)	0.505[d]	1.000
No	61 (14.4)	362 (85.6)		
**Exhaustion (n=425)**				
Yes	5 (25.0)	15 (75.0)	2.050[d]	0.180
No	55 (13.6)	350 (86.4)		
**Stroke (n=426)**				
Yes	0	1 (100.0)	0.168[d]	1.000
No	61(14.4)	364 (85.6)		
**Mental illness (n=426)**				
Yes	1 (50.0)	1 (50.0)	2.085[d]	0.266
No	60 (14.2)	364 (85.8)		
**Other chronic illnesses (n=426)**				
Yes	7 (31.8)	15 (68.2)	5.790[d]	0.026
No	54 (13.4)	350 (86.6)		
**Stressful life event (n=430)**				
No	16 (7.7)	192 (92.3)	15.602[a]	< 0.001
Yes	175 (78.8)	175 (78.8)		
**Assault (n=430)**				

No	53 (13.1)	352 (86.9)	13.640[d]	0.001
Yes	10 (40.0)	15 (60.0)		
**Prolonged serious illness (n=430)**				
No	52 (13.1)	346 (86.9)	10.756[d]	0.003
Yes	11 (34.4)	21 (65.6)		
**Bullied during childhood (n=430)**				
No	55 (13.6)	349 (86.4)	5.749[d]	0.038
Yes	8 (30.8)	18 (69.2)		
**Injury due to accident (n=430)**				
No	52 (13.1)	344 (86.9)	9.252[d]	0.009
Yes	11 (32.4)	23 (67.6)		
**Orphan below 10 years old (n=430)**				
No	60 (14.3)	359 (85.7)	1.438[d]	0.209
Yes	3 (27.3)	8 (72.7)		
**Loss of loved one (n=430)**				
No	36 (12.9)	244 (87.1)	2.066[a]	0.151
Yes	27 (18.0)	123(82.0)		
**Marital issue (n=430)**				
No	51 (12.5)	357 (87.5)	29.511[d]	< 0.001
Yes	12 (54.5)	10 (45.5)		
**Family issue (n=430)**				
No	49 (12.2)	353 (87.8)	29.928[a]	< 0.001
Yes	14 (50.0)	14 (50.0)		
**Financial issue (n=430)**				
No	49 (12.1)	355 (87.9)	33.998[d]	< 0.001
Yes	14 (53.8)	12 (46.2)		
**Neighborhood issue (n=430)**				
No	56 (13.6)	355 (86.4)	7.828[d]	0.012
Yes	7 (36.8)	12 (63.2)		
**Working environment issue (n=430)**				
No	44 (11.5)	33 (88.5)	26.859[a]	< 0.001
Yes	19 (39.6)	29 (60.4)		
**Loss of job (n=430)**				
No	47 (12.3)	335 (87.7)	15.081[a]	< 0.001
Yes	16 (33.3)	32 (66.7)		
**Legal issue (n=430)**				
No	57 (13.8)	356 (86.2)	6.032[d]	0.026
Yes	6 (35.3)	11 (64.7)		
**Substance use (n=430)**				
No	40 (13.3)	260 (86.7)	1.378[a]	0.240
Yes	23 (17.7)	107 (82.3)		

**Smoking (n=430)**				
No	44 (14.0)	270 (86.0)	0.379[a]	0.538
Yes	19 (16.4)	97 (83.6)		
**Alcohol (n=430)**				
No	56 (14.1)	341 (85.9)	1.230[d]	0.302
Yes	7 (21.2)	26 (78.8)		
**Cannabis (n=430)**				
No	62 (14.5)	365 (85.5)	0.843[d]	0.379
Yes	1 (33.3)	2 (66.7)		
**Cocaine (n=430)**				
No	63 (14.7)	366 (85.3)	0.172[d]	1.000
Yes	0	1 (100.0)		
**Amphetamine-related (n=430)**				
No	63 (14.7)	366 (85.3)	0.172[d]	1.000
Yes	0	1 (100.0)		
**Inhaler (n=430)**				
No	63 (14.7)	365 (85.3)	0.345[d]	1.000
Yes	0	2 (100.0)		
**Sleeping pill (n=430)**				
No	58 (13.7)	364 (86.3)	14.925[d]	0.002
Yes	5 (62.5)	3 (37.5)		
**Hallucinogen (n=430)**				
No	62 (14.5)	366 (85.5)	2.008[d]	0.272
Yes	1 (50.0)	1 (50.0)		
**Opioid (n=429)**				
No	63 (14.7)	365 (85.3)	0.173[d]	1.000
Yes	0	1 (100.0)		
**Others (n=429)**				
No	62 (14.6)	636 (85.4)	0.343[d]	0.472
Yes	1 (25.0)	3 (75.0)		

[a] = Pearson Chi-square; [b]= Independent t-test; [c]= Mann-Whitney U test; [d] = Fischer's exact test.

Table F. Univariable analysis on barriers to help seeking associated with anxiety (symptomatic) among B40 respondents from the Petaling district (n=432)

Barriers to help seeking	Anxiety (symptomatic)		Test statistic	*p* value
	≥ 5	< 5		
**Overall average poverty attribution (n=430)**	3.48 (0.86)	3.43 (0.86)	11285.000[c]	0.762
**Structural (average) (n=430)**	3.40 (1.00)	3.80 (1.20)	11993.500[c]	0.036
**Socioeconomic (average) (n=430)**	4.00 (0.80)	3.80 (1.20)	11218.500[c]	0.706
**Individualistic (average) (n=430)**	3.60 (1.30)	3.60 (1.60)	11424.000[c]	0.880
**Fatalistic (average) (n=430)**	3.00 (1.20)	3.00 (1.10)	11413.500[c]	0.872
**Health literacy (average) (n=427)**	2.83 (1.00)	3.00 (0.83)	9105.000[c]	0.008
**Health literacy index (n=427)**	30.56 (16.67)	33.33 (13.89)	9105.000[c]	0.008
**Help seeking (total) (n=425)**	52.00 (12.50)	55.00 (18.00)	9434.500[c]	0.057
**Help seeking (average) (n=425)**	5.78 (1.39)	6.11 (2.00)	9434.500[c]	0.057
**Self-stigma (total) (n=429)**	28.00 (5.00)	25.00 (8.00)	7843.500[c]	< 0.001
**Self-stigma (average) (n=429)**	2.80 (0.50)	2.50 (0.80)	7843.500[c]	< 0.001
**Resilience (total) (n=428)**	63.00 (20.50)	73.00 (20.00)	7452.000[c]	< 0.001
**Resilience categorical (n=428)**				
Low	31 (23.5)	101 (76.5)	16.485[a]	< 0.001
Moderate	23 (14.2)	139 (85.8)		
High	8 (6.0)	126 (94.0)		

[a] = Pearson Chi-square; [b]= Independent t-test; [c]= Mann-Whitney U test.

Table G. Univariable analysis on socio-demographic factors associated with EQ-5D among B40 respondents from the Petaling district (n=432)

Socio-demographic variables	EQ-5D		Test statistic	*p* value
	Any problem	No problem		
**Gender (n=430)**				
Male	67 (24.2)	210 (75.8)	4.728[a]	0.030
Female	52 (34.0)	101 (66.0)		
**Age (n=412)**	47.3 (14.1)	41.5 (12.5)	-4.014[b]	<0.001
**Age category (n=412)**				
< 30	12 (18.5)	53 (81.5)	19.060[a]	< 0.001
30-40	26 (19.8)	105 (80.2)		
41-50	27 (25.7)	78 (74.3)		
> 50	47 (42.3)	64 (57.7)		
**Ethnicity (n=429)**				
Malay	85 (26.4)	237 (73.6)	4.072[a]	0.254
Chinese	14 (24.1)	44 (75.9)		
Indian	13 (41.9)	18 (58.1)		
Others	6 (33.3)	12 (66.7)		
**Marital status (n=429)**				
Single/Divorced	18 (24.0)	57 (76.0)	7.234[a]	0.027
Widowed	16 (47.1)	18 (52.9)		
Married	84 (26.3)	236 (73.8)		
**Education level (n=426)**				
No formal Education and Primary	15 (51.7)	14 (48.3)	9.050[a]	0.011
Secondary school	55 (26.8)	150 (73.2)		
>Secondary	48 (25.1)	143 (74.9)		
**Employment status before outbreak (n=425)**				
Yes	91 (25.6)	265 (74.4)	5.306[a]	0.021
No	27 (39.1)	42 (60.9)		
**Employment status during outbreak (n=412)**				
Yes	60 (21.8)	215 (78.2)	14.150[a]	< 0.001
No	54 (39.4)	83 (60.6)		
**Household income before outbreak (n=431)**	3000.0 (2400.0)	3000.0 (2400.0)	17511.000[c]	0.362
**Household income before outbreak groups (n=431)**				
<RM 1700	28 (32.6)	58 (67.4)	2.025[a]	0.846
RM 1700 to 2700	28 (28.3)	71 (71.7)		
RM 2701 to 3700	25 (25.8)	72 (74.2)		
RM 3701 to 4700	14 (22.6)	48 (77.4)		
RM 4700-5700	10 (27.8)	26 (72.2)		
>RM 5701	14 (27.5)	37 (72.5)		

**Household income during outbreak (n=431)**	2600.0 (2684.0)	2558.0 (2600.0)	17743.000[c]	0.477
**Household income during outbreak groups (n=431)**				
<RM 1700	41 (30.4)	94 (69.6)	4.090[a]	0.537
RM 1700 to 2700	22 (24.2)	69 (75.8)		
RM 2701 to 3700	22 (28.6)	55 (71.4)		
RM 3701 to 4700	18 (32.7)	37 (67.3)		
RM 4700-5700	9 (28.1)	23 (71.9)		
>RM 5701	7 (17.1)	34 (82.9)		
**Household size (n=424)**				
< 4	51 (26.7)	140 (73.3)	0.075[a]	0.784
≥ 4	65 (27.9)	168 (72.1)		
**Years-live in residential area (n=399)**				
≤ 4 years	20 (25.6)	58 (74.4)	0.100[a]	0.752
> 4 years	88 (27.4)	233 (72.6)		
**House-ownership (n=429)**				
Owners/Inherited	59 (29.9)	138 (70.1)	1.091[a]	0.296
Shelter/Renting	59 (25.4)	173 (74.6)		
**Asset- House (n=429)**				
Yes	65 (28.1)	166 (71.9)	0.081[a]	0.776
No	53 (26.9)	144 (73.1		
**Asset-Vehicle (n=429)**				
Yes	90 (25.8)	259 (74.2)	2.770[a]	0.096
No	28 (35.0)	52 (65.0)		
**Asset-Land (n=427)**				
Yes	18 (42.9)	24 (57.1)	5.398[a]	0.020
No	100 (26.0)	285 (74.0)		
**Asset-Orchard (n=427)**				
Yes	9 (39.1)	14 (60.9)	1.606[a]	0.205
No	109 (27.0)	295 (73.0)		
**Asset-Cash (n=428)**				
Yes	32 (21.6)	116 (78.4)	4.009[a]	0.045
No	86 (30.7)	194 (69.3)		
**Asset-Rental house (n=427)**				
Yes	20 (36.4)	35 (63.6)	2.405[a]	0.121
No	98 (26.3)	274 (73.7)		
**Asset-Jewelry (n=427)**				
Yes	37 (28.9)	91 (71.1)	0.148[a]	0.701
No	81 (27.1)	218 (72.9)		
**Asset-Shophouse (n=427)**				
Yes	1 (16.7)	5 (83.3)	0.366[d]	1.000
No	117 (27.8)	304 (72.2)		
**Asset-Investment (n=427)**				
Yes	11 (22.4)	38 (77.6)	0.744[a]	0.388

No	107 (28.3)	271 (71.7)		
**Asset-Others (n=426)**				
Yes	0	4 (100.0)	1.529[d]	0.579
No	117 (27.7)	305 (72.3)		
**Communication tools**				
**Own mobile or smartphone**				
Yes	94 (27.3)	245(72.3)	[a]0.2615	0.609
No	23(30.7)	52(69.3)		
**Own at least a form of communication tool (TV Or Radio and Internet or laptop and Smartphone/ mobile)**				
Yes	113(28.0)	291(72.0)	0.697 [d]	0.303
No	4 (40.0)	6(60.0)		
**Religiosity (n=431)**	17.0 (11.0)	18.0 (5.0)	16817.500[c]	0.126
**Religiosity category (n=431)**				
Low (<17)	54 (29.7)	138 (70.3)	0.669[a]	0.413
High (≥17)	65 (26.1)	184 (73.9)		
**Community connectedness (n=430)**				
once a week	71 (25.2)	211 (74.8)	2.552[a]	0.110
< once a month	48 (32.4)	100 (67.6)		

[a] = Pearson Chi-square; [b]= Independent t-test; [c]= Mann-Whitney U test; [d] = Fischer's exact test.

Table H. Univariable analysis on health-related profiles associated with EQ-5D among B40 respondents from the Petaling district (n=432)

Health-related variables	EQ-5D		Test statistic	*p* value
	Any problem	No problem		
**BMI (kg/m^2) (n=429)**	25.5 (6.5)	24.5 (5.3)	16358.000[c]	0.083
**BMI categories (n=429)**				
<18.5 kg/m2	6 (20.7)	23 (79.3)	3.835[d]	0.444
18.5-22.9 kg/m2	25 (26.0)	71 (74.0)		
23.0-27.4 kg/m2	45 (26.2)	127 (73.8)		
27.5- 34.9 kg/m2	32 (29.9)	75 (70.1)		
≥ 35.0 kg/m2	8 (44.4)	10 (55.6)		
**History of chronic illnesses (n=427)**				
Yes	49 (16.4)	249 (83.6)	57.326[a]	<0.001
No	67 (51.9)	62 (48.1)		
**Hypertension (n=427)**				
Yes	41 (53.2)	36 (46.8)	32.294[a]	<0.001
No	75 (21.4)	275 (78.6)		
**Diabetes Mellitus (n=427)**				
Yes	24 (64.9)	13 (35.1)	29.097[a]	<0.001
No	92 (23.6)	198 (76.4)		
**Heart disease (n=427)**				
Yes	12 (80.0)	3 (20.0)	21.932[d]	<0.001
No	104 (25.2)	308 (74.8)		
**Kidney disease (n=427)**				
Yes	7 (100.0)	0	19.080[d]	<0.001
No	109 (26.0)	311 (74.0)		
**Cancer (n=427)**				
Yes	3 (100.0)	0	8.100[d]	0.020
No	113 (26.7)	311 (73.3)		
**Exhaustion (n=426)**				
Yes	7 (35.0)	13 (65.0)	0.682[a]	0.409
No	108 (26.6)	298 (73.4)		
**Stroke (n=427)**				
Yes	1 (100.0)	0	2.687[d]	0.272
No	115 (27.0)	311 (73.0)		
**Mental illness (n=427)**				
Yes	2 (100.0)	0	5.387[d]	0.073
No	114 (26.8)	311 (73.2)		
**Other chronic illnesses (n=427)**				
Yes	15 (68.2)	7 (31.8)	19.721[a]	<0.001
No	101 (24.9)	304 (75.1)		
**Stressful life event (n=430)**				
Yes	84 (37.8)	138 (62.2)	23.685[a]	<0.001
No	35 (16.8)	173 (83.2)		

**Assault (n=430)**				
Yes	14 (56.0)	11 (44.0)	10.640[a]	0.001
No	105 (25.9)	300 (74.1)		
**Prolonged serious illness (n=430)**				
Yes	20 (62.5)	12 (37.5)	20.949[a]	<0.001
No	99 (24.9)	299 (75.1)		
**Bullied during childhood (n=430)**				
Yes	10 (38.5)	16 (61.5)	1.609[a]	0.205
No	109 (27.0)	295 (73.0)		
**Injury due to accident (n=430)**				
Yes	17 (50.0)	17 (50.0)	9.194[a]	0.002
No	102 (25.8)	294 (74.2)		
**Orphan below 10 years old (n=430)**				
Yes	7 (63.6)	4 (36.4)	7.294[d]	0.013
No	112 (26.7)	307 (73.3)		
**Loss of loved one (n=430)**				
Yes	58 (38.7)	92 (61.3)	13.906[a]	<0.001
No	61 (21.8)	219 (78.2)		
**Marital issue (n=430)**				
Yes	13 (59.1)	9 (40.9)	11.433[a]	0.001
No	106 (26.0)	302 (74.0)		
**Family issue (n=430)**				
Yes	14 (50.0)	14 (50.0)	7.458[a]	0.006
No	105 (26.1)	297 (73.9)		
**Financial issue (n=430)**				
Yes	18 (69.2)	8 (30.8)	23.876[a]	<0.001
No	101 (25.0)	303 (75.0)		
**Neighborhood issue (n=430)**				
Yes	10 (52.6)	9 (47.4)	6.186[a]	0.013
No	109 (26.5)	302 (73.5)		
**Working environment issue (n=430)**				
Yes	17 (35.4)	31 (64.6)	1.618[a]	0.203
No	102 (26.7)	280 (73.3)		
**Loss of job (n=430)**				
Yes	22 (45.8)	26 (54.2)	8.901[a]	0.003
No	97 (25.4)	285 (74.6)		
**Legal issue (n=430)**				
Yes	8 (47.1)	9 (52.9)	3.323[d]	0.094
No	111 (26.9)	302 (73.1)		
**Substance use (n=430)**				
Yes	35 (26.9)	95 (73.1)	0.053[a]	0.819

No	84 (28.0)	216 (72.0)		
**Smoking (n=430)**				
Yes	34 (29.3)	82 (70.7)	0.212[a]	0.645
No	85 (27.1)	229 (72.9)		
**Alcohol (n=430)**				
Yes	10 (30.3)	23 (69.7)	0.123[a]	0.725
No	109 (27.5)	288 (72.5)		
**Cannabis (n=430)**				
Yes	1 (33.3)	2 (66.7)	0.048[d]	1.000
No	118 (27.6)	309 (72.4)		
**Cocaine (n=430)**				
yes	0	1 (100.0)	0.384[d]	1.000
No	119 (27.7)	310 (72.3)		
**Amphetamine-related (n=430)**				
Yes	0	1 (100.0)	0.384[d]	1.000
No	119 (27.7)	310 (72.3)		
**Inhaler (n=430)**				
Yes	0	2 (100.0)	0.769[d]	1.000
No	119 (27.8)	309 (72.2)		
**Sleeping pill (n=430)**				
Yes	5 (62.5)	3 (37.5)	4.939[d]	0.040
No	114 (27.0)	308 (73.0)		
**Hallucinogen (n=430)**				
Yes	1 (50.0)	1 (50.0)	0.500[d]	0.477
No	118 (27.6)	310 (72.4)		
**Opioid (n=429)**				
Yes	0	1 (100.0)	0.385[d]	1.000
No	119 (27.8)	309 (72.2)		
**Others (n=429)**				
Yes	1 (25.0)	3 (75.0)	0.015[d]	1.000
No	118 (27.8)	307 (72.2)		
**Depression (n=428)**	4.0 (6.0)	2.0 (3.0)	12744.000[c]	<0.001
**Depression-diagnosis (n=428)**				
<10	101 (25.4)	297 (74.6)	16.660[a]	<0.001
≥10	18 (60.0)	12 (40.0)		
**Depression-symptomatic (n=428)**				
<5	61 (20.3)	240 (79.7)	28.714[a]	<0.001
≥5	58 (45.7)	69 (54.3)		
**Anxiety (n=429)**	2.0 (5.0)	1.0 (2.0)	13098.500[c]	<0.001
**Anxiety-diagnosis (n=429)**				
<8	104 (25.4)	306 (74.6)	26.009[a]	<0.001
≥8	15 (78.9)	4 (21.1)		

**Anxiety-symptomatic (n=429)**				
<5	83 (22.7)	283 (77.3)	31.852[a]	<0.001
≥5	36 (57.1)	27 (42.9)		

[a] = Pearson Chi-square; [b]= Independent t-test; [c]= Mann-Whitney U test; [d] = Fischer's exact test.

Table I. Univariable analysis on barriers to help seeking associated with EQ-5D among B40 respondents from the Petaling district (n=432)

Barriers to help seeking	EQ-5D		Test statistic	*p* value
	Any problem	No problem		
**Overall average poverty attribution (n=430)**	3.4 (0.6)	3.4 (0.7)	0.055[b]	0.956
**Structural (average) (n=430)**	3.4 (0.9)	3.6 (0.90)	2.017[b]	0.044
**Socioeconomic (average) (n=430)**	3.8 (0.7)	3.8 (0.9)	-0.100[b]	0.920
**Individualistic (average) (n=430)**	3.3 (1.0)	3.4 (1.1)	0.754[b]	0.451
**Fatalistic (average) (n=430)**	3.2 (0.9)	3.0 (1.0)	-2.022[b]	0.044
**Health literacy (average) (n=428)**	2.8 (0.7)	3.1 (0.6)	4.393[b]	<0.001
**Health literacy index (n=428)**	29.8 (11.3)	34.8 (10.2)	4.393[b]	<0.001
**Help seeking (total) (n=425)**	53.0 (20.5)	55.0 (17.0)	15748.500[c]	0.057
**Help seeking (average) (n=425)**	5.9 (2.3)	6.1 (1.9)	15748.500[c]	0.057
**Self-stigma (total) (n=429)**	26.4 (5.3)	24.3 (5.9)	-3.412	0.001
**Self-stigma (average) (n=429)**	2.6 (0.5)	2.4 (0.6)	-3.401	0.001
**Resilience (total) (n=428)**	69.06 (13.79)	74.70 (14.79)	3.589	< 0.001
**Resilience categorical (n=428)**				
Low	45 (33.8)	88 (66.2)	9.238[a]	0.010
Moderate	49 (30.2)	113 (69.8)		
High	24 (18.0)	109 (82.0)		

[a] = Pearson Chi-square; [b]= Independent t-test; [c]= Mann-Whitney U test.

Model fitting using Hosmer-Lemeshow approach

Table J. Logistic regression model fitting using Hosmer-Lemeshow approach (PHQ_Symptomatic)

Model	-2loglikelihood	df	Note	Diff in -2loglikelihood	Diff in df	Critical value	Comment
1	310.692	41	Saturated/Full model	-	-	-	-
2	310.864	40	Dropped BMI	0.172	1	3.841	<
3	310.864	39	Dropped PA_Fatalistic	0.000	1	3.841	<
4	313.628	34	Dropped House income before Covid-19	2.764	5	4.352	<
5	313.684	33	Dropped SLE_13_3	0.056	1	3.841	<
6	313.706	32	Dropped Penyakit_Jantung	0.022	1	3.841	<
7	313.828	31	Dropped SLE_13_6	0.122	1	3.841	<
8	313.968	30	Dropped SCSORF_Total	0.140	1	3.841	<
9	314.150	29	Dropped PA_Individualistic	0.182	1	3.841	<
10	314.418	28	Dropped SLE_13_13	0.268	1	3.841	<
11	314.690	27	Dropped Aset_Rumahsewa	0.272	1	3.841	<
12	314.990	26	Dropped SLE_13_12	0.300	1	3.841	<

13	315.204	25	Dropped ASSIST_1_g	0.214	1	3.841	<
14	315.456	24	Dropped Community_connectedness	0.252	1	3.841	<
15	315.890	23	Dropped SLE_13_10	0.434	1	3.841	<
16	316.438	22	Dropped SLE_13_4	0.548	1	3.841	<
17	317.296	21	Dropped Buah_pinggang	0.858	1	3.841	<
18	318.284	20	Dropped ASSIST_1_b	0.988	1	3.841	<
19	319.984	19	Dropped SLE_13_7	1.700	1	3.841	<
20	322.068	18	Dropped ASSIST_1_a	2.084	1	3.841	<
21	324.430	17	Dropped SLE_13_9	2.358	1	3.841	<
22	326.946	16	Dropped SSOSH_T	2.516	1	3.841	<
23	329.334	15	Dropped Average_HL	2.388	1	3.841	<
24	333.814	14	Dropped Aset_RumahKedai (retained)	4.480	1	3.841	<
25	338.000	13	Dropped education_cat	5.000	1	3.841	<

Table K. Logistic regression model fitting using Hosmer-Lemeshow approach (GAD_Symptomatic)

Model	-2loglikelihood	df	Note	Diff in -2loglikelihood	Diff in df	Critical value	Comment
1	228.530	37	Saturated/Full model	-	-	-	-
2	228.699	36	Dropped HL	0.169	1	3.841	<
3	228.716	35	Dropped SLE_13_4	0.017	1	3.841	<
4	228.983	34	Dropped House ownership	0.267	1	3.841	<
5	229.060	33	Dropped SLE 13 8	0.077	1	3.841	<
6	229.159	32	Dropped SLE 13 13	0.099	1	3.841	<
7	236.959	31	Dropped MHSAS_T (retained)	7.800	1	3.841	>
8	229.422	31	Dropped Heart disease	0.263	1	3.841	<
9	229.650	30	Dropped other diseases	0.228	1	3.841	<
10	229.913	29	Dropped vehicle asset	0.263	1	3.841	<
11	230.328	28	Dropped SCSORF_T	0.415	1	3.841	<
12	230.677	27	Dropped SLE 13 12	0.349	1	3.841	<
13	231.070	26	Dropped SLE 13 10	0.393	1	3.841	<
14	231.612	25	Dropped SLE 13 6	0.542	1	3.841	<

15	232.034	24	Dropped SLE 13 3	0.422	1	3.841	<
16	232.436	23	Dropped Hypertension	0.402	1	3.841	<
17	242.466	20	Dropped Age (retained)	10.03	3	7.815	>
18	233.148	22	Dropped SLE 13 2	0.712	1	3.841	<
19	234.238	21	Dropped SLE 13 1	1.090	1	3.841	<
20	235.628	20	Dropped Community connectedness	1.390	1	3.841	<
21	238.000	19	Dropped SLE 13 5	2.372	1	3.841	<
22	249.504	14	Dropped BMI	8.637	4	9.488	<
23	251.747	13	Dropped kidney disease	2.243	1	3.841	<
24	256.104	12	Dropped rental house asset retained	4.357	1	3.841	>
25	300.00	11	Dropped education_cat	4.000	1	3.841	<

Table L. Logistic regression model fitting using Hosmer-Lemeshow approach (EQ-5D)

Model	-2loglikelihood	df	Note	Diff in -2loglikelihood	Diff in df	Critical value	Comment
1	294.518	42	Saturated/Full model	-	-	-	-
2	295.872	40	Dropped Marital status	1.354	2	5.991	<
3	295.876	39	Dropped asset-vehicle	0.004	1	3.841	<
4	295.914	38	Dropped PA-Fatalistic	0.038	1	3.841	<
5	297.833	37	Dropped MHSAS-mean	1.919	1	3.841	<
6	297.932	36	Dropped SLE_13_11	0.099	1	3.841	<
7	298.056	35	Dropped Community connectedness	0.124	1	3.841	<
8	298.197	34	Dropped PA-Structural	0.141	1	3.841	<
9	298.329	33	Dropped GDA-symptomatic	0.132	1	3.841	<
10	298.749	31	Dropped work before Covid-19	0.256	1	3.841	<
11	298.953	30	Dropped asset-Ladang	0.204	1	3.841	<
12	299.292	29	Dropped SLE_13_10	0.339	1	3.841	<
13	299.670	28	Dropped SLE_13_8	0.378	1	3.841	<
14	300.165	27	Dropped SLE_13_2	0.495	1	3.841	<

15	300.954	26	Dropped Religiosity	0.789	1	3.841	<
16	301.884	25	Dropped SLE_13_7	0.930	1	3.841	<
17	302.557	24	Dropped SLE_13_1	0.673	1	3.841	<
18	305.044	22	Dropped RC_3CAT	2.487	2	5.991	<
19	306.638	21	Dropped Assist-g	1.594	1	3.841	<
20	308.151	20	Dropped SSOSH-mean	1.513	1	3.841	<
21	322.092	19	Dropped work during covid (reserved)	13.941	1	3.841	>
22	309.831	19	Dropped Heart disease	1.680	1	3.841	<
23	311.390	18	Dropped SLE_13_3	1.559	1	3.841	<
24	313.273	17	Dropped Aset-Tanah	1.893	1	3.841	<
25	319.260	16	Dropped SLE_13_13	3.001	1	3.841	<
26	340.463	15	Dropped age Cat (reserved)	21.203	1	3.841	>
27	322.054	15	Dropped SLE_13_9	2.794	1	3.841	<
28	325.567	14	Dropped SLE_13_6	3.513	1	3.841	<
29	328.792	13	Dropped Rumah Sewa	3.225	1	3.841	<

30	332.149	12	Dropped SLE_13_5	3.357	1	3.841	<
31	339.00	11	Dropped education_cat	7.000	1	3.841	<

Note: After running 32 models in purposive variables selection using Hosmer-Lemeshow approach, only 10 variables remained
