# Supplementary material for: Psychosocial factors associated with mental health and quality of life during the COVID-19 pandemic among low-income urban dwellers in Peninsular Malaysia
Source: PLoS One. 2022 Aug 23;17(8):e0264886. doi: 10.1371/journal.pone.0264886 (PMC9398022; doi:10.1371/journal.pone.0264886)
Supplement: Attachment — Submitted filename: PONE-D-22-05096R1 comments 27June2022.docx [file pone.0264886.s006.docx]

1. Abstract.

Conclusion. I don't think using words 'higher proportion' compared to 'pre-pandemic reports' can be used in conclusions. There is no information on pre-pandemic reports in background or results. Therefore, if a reader is only looking through the abstract without reading the whole paper, they would not come to this conclusion.

Track-changes document

2. Introduction

Lines 63-64. Authors state the increase in suicide cases from 609 in 2019 to 631 in 2020. Was there also an increase in country’s population (and thus no increase in suicide rates per capita) over that time?

Line 85. Please provide description or an example of ‘structural issues’.

Line 92. ‘Financial loss’ and ‘job loss’ used in the same line. Suggest to change wording to avoid repetition.

Line 93. ‘…financial issues are important stressors that predict depression’ – suggest change wording to ‘can lead to’ or similar.

Lines 93 – 99. Very long and confusing sentence. Suggest you split it in two.

Line 95. ‘…stringent government containment measures represents a protective factor for depression’ – what’s the mechanism? How is it a protective factor? (reduces fear of getting sick and being hospitalised?)

Line 95. ‘…this should not delay any public…’ What is ‘this’ referring to?

3. Methods

Line 138. How the data on psychiatric illness was obtained?

Line 139. The whole household where non-Malaysians lived was excluded?

Line 144. ‘…Since this is a face-to-face…’ – change to was. Keep your past and present tense consistent throughout.

4. Results

Line 247-248. Of a total 504 eligible participants, 432 (82.7%) completed the survey.

Table 1.

I cannot find household income before Covid-19 in this table.

Secondary schools and more than secondary schools % are in brackets. Is there a reason why this is different to other data presented in this table?

Employed: yes/no or employment status: employed/not employed. ‘Employment status’ does not define if you are or are not employed.

Employment status (during Covid-19). Change the order of yes and no lines so it matches the order of the employment status (before Covid-19).

Household size. =4 belongs to which bracket?

House ownership. What is inherited mean? Aren’t they home owners?

Lines 256-257. Did you mean ‘Structural and socio-economic domains scores of poverty attribution were…’? It reads as if you are providing scores for three domains.

Lines 262 – 264. Not clear. Is this 30% of male respondents or 30% of all participants? 130 is a third of your entire cohort, but this reads as if you are talking about male respondents only.

n=278 male

130/278 (xxx%) used substances

Line 264-265. Is this sentence still describing male participants only?

Line 266. ‘(n=30)’ Is this a typo?

Line 272-279. age less than 30... is a variable

‘those aged less than 30’ refers to participants.

You talking about participants not variables here. This applies to first two sentences in this paragraph. Try to re-phrase if you want to write about factors.

Table 2.

Sleep pill usage. ‘Reference’ duplication.

Describe all the abbreviations used in this table.

5. Discussion

Line 319-320. Suggest use 'respondents' here instead of group and delete word 'respondents' after 'financial strain'.

Line 332. It would be good to see some numbers found in studies you're including in your reference index to support your statement.

Line 338. Delete ‘were’.

Line 388. ’...to the mid-20s to the 30s of age’ – fix wording

Line 434. Cardiovascular problems? Adverse cardiovascular outcomes? Use something similar instead just ‘cardiovascular outcomes’.

Line 466. Not sure what you mean by ‘anxiety symptoms but not for depression’

Line 469-470. Think about re-writing to avoid repetitions

Line 477. Delete ‘a’ in ‘…of health a promotion’

Line 494. Did you mean ‘…structural issues were more likely to experience…’

Line 538-539. One third had low resilience and higher resilience scores. Isn't is mutually exclusive?

Line 539-540. ‘Substantial empirical studies have supported the inverse resilience scores concerning depression and anxiety’. Not sure what this sentence means.
