# Supplementary material for: Psychosocial factors associated with mental health and quality of life during the COVID-19 pandemic among low-income urban dwellers in Peninsular Malaysia
Source: PLoS One. 2022 Aug 23;17(8):e0264886. doi: 10.1371/journal.pone.0264886 (PMC9398022; doi:10.1371/journal.pone.0264886)
Supplement: Attachment — Submitted filename: Review for Manuscript Number PONE.docx [file pone.0264886.s007.docx]

**Review for Manuscript Number PONE-D-22-05096**
"Psychosocial factors associated with mental health and quality of life during the COVID-19 pandemic among low-income urban dwellers in Peninsular Malaysia Psychosocial factors for mental health among the low-income community"

Abstract:

Clearly written and concise.

Introduction

Presented relevantly.

Methods

Clearly presented.

However, regarding two of the tools used:

1. EQ-5D
2. SCSRFQ

As mentioned in the manuscript (line 134: The respondents completed multiple standardized instruments in the Malay language with assistance from the research team), were the tools mentioned above translated and validated in Malay language for this study? The other tools were clearly informed with reference regarding its validity of Malay language questionnaires.

Statistical Analysis:

Not an expert to comment on this but the presentation of data was clear and easily understood.

Results:

No comment

Discussion:

It addresses the results adequately and well thought through. Findings are highlighted with relevant suggestions on the importance of mental health and psychosocial interventions that can be taken up to policymakers.

Limitations were addressed.
