# Supplementary material for: Psychosocial factors associated with mental health and quality of life during the COVID-19 pandemic among low-income urban dwellers in Peninsular Malaysia
Source: PLoS One. 2022 Aug 23;17(8):e0264886. doi: 10.1371/journal.pone.0264886 (PMC9398022; doi:10.1371/journal.pone.0264886)
Supplement: Attachment — Submitted filename: Response to Reviewers1.docx [file pone.0264886.s008.docx]

**Academic Editor**

**Journal Requirements:**

Please review your reference list to ensure that it is complete and correct. If you have cited papers that have been retracted, please include the rationale for doing so in the manuscript text, or remove these references and replace them with relevant current references. Any changes to the reference list should be mentioned in the rebuttal letter that accompanies your revised manuscript. If you need to cite a retracted article, indicate the 'article's retracted status in the References list and include a citation and full reference for the retraction notice.

***Response:***

***The reference list was cross-checked and updated. New references were added as below :***

48. Mohamad AS, Draman S, Aris MAM, Musa R, Rus RM, Malik M. Depression, anxiety, and stress among adolescents in Kuantan and its association with religiosity-a pilot study. IIUM Medical Journal Malaysia. 2018;17(2):92-5.

**Reviewers**

**Reviewer #2:** I recommend the study "Psychosocial factors associated with mental health and quality of life during the

COVID-19 pandemic among low-income urban dwellers in Peninsular Malaysia

Psychosocial factors for mental health among the low-income community" for publication.

- ***Response :*** *Thank you for your suggestion.*

**Reviewer #3:** Data availability statement: Data cannot be shared publicly because of the vulnerability of the population. Was data anonymised? How was the data stored and managed? De-identified data would not compromise the subjects.

Minor comments attached.

- ***Response:*** *Thank you for your questions.*

*The data is anonymised and stored according to the standard protocol stipulated in the data management and storage guidelines from University Malaya. The data will be available upon request by other interested researchers.*

**Reviewer #4:** I was wondering during the MCO (September-November 2020) when the research was done face to face, were the research welcomed to the subjects' homes or was there any resistance faced? As we know, at that point, movements were still restricted so was it ethical to conduct this study face-to face? Would it not have increased the risk of infection? How was this addressed?

- ***Response:*** *Thank you for your questions. September to early November 2020 were within the period of Recovery of Movement Control Order (RMCO) in Malaysia. As COVID-19 cases were successful under control in this country, the SOP was relaxed, giving the window of opportunity for the research team to conduct the face-to-face data collection with permission from the Faculty of Medicine. Therefore, the team did not face many issues during the data collection. Nevertheless, the Principle Investigator had obtained approval from the Dean's office of the Faculty of Medicine to conduct the data collection strictly with COVID-19 SOP. Enumerators were given a proper briefing on the fieldwork COVID-19 protocol from the University Malaya before the commencement of the data collection.*

**Responses to the email attachments (Words Documents)**

**Reviewer # xx**

1. Abstract.
a) Conclusion. I don't think using words 'higher proportion' compared to 'pre-pandemic reports' can be used in conclusions. There is no information on pre-pandemic reports in the background or results. Therefore, if a reader is only looking through the abstract without reading the whole paper, they would not come to this conclusion.

- **Response: Thank you for your response. The statement has been revised as below:**

*“A high proportion of mild to severe anxiety and depression symptoms was reported in the sampled urban poor population.*”(*Line 51 -52)*

**Track-changes document**

2. Introduction
a) Lines 63-64. Authors state the increase in suicide cases from 609 in 2019 to 631 in 2020. Was there also an increase in 'country's population (and thus no increase in suicide rates per capita) over that time?

- **Response*: Thank you for your comments. We have implemented the changes as below:***

*“*However*, since there was an increase in country's population, thus no increase in suicide rates per capita was observed.” (Line 63-65)*

b) Line 85. Please provide description or an example of 'structural 'issues'.

- **Response*: Thank you for your responses, based on the reference by Mickelson et al. 2014, the description of 'structural 'issues' has been added into the statement:***

*"Additionally, low-income individuals' perceptions of the structural issues, attributions related to government blame or discrimination, may subsequently influence their decision-making and mental health.”(Line 84-86)*

c) Line 92. 'Financial 'loss' and 'job 'loss' used in the same line. Suggest changing wording to avoid repetition.

- ***Response: Thank you for your comments. We have implemented the changes as below:***

*"*Undoubtedly*, the government's containment measures prevented the spread of the coronavirus, but a significant number of people suffered from financial loss due to job dismissal and pay cuts.” (Line 90-92)*

d) Line 93. ''…financial issues are important stressors that predict 'depression' – suggest change wording to 'can lead 'to' or similar.

- **Response*:* Thank you for your comments. We have implemented the changes as below:**

*"*Unemployment *and financial issues are important stressors that can lead to depression.” (Line 92-93)*

e) Lines 93 – 96. Very long and confusing sentence. Suggest you split it in two.

- ***Response: Improvement has been done as shown below:***

*"Even though evidence showed stringent government containment measures moderate depression by promoting trust and ease uncertainty (30), timely implementation of early screening and intervention for mental health at-risk individuals should be in place to prevent severe COVID-19 cases. A recent meta-analysis suggests that individuals with pre-existing mood disorders are at higher risk of COVID-19 hospitalisation and death (31)."(Line 93-96).*

f) Line 95. ''…stringent government containment measures represents a protective factor for 'depression' – 'what's the mechanism? How is it a protective factor? (reduces fear of getting sick and being hospitalised?)

- ***Response: Improvement has been done and shown below:***

***"****Even though evidence showed stringent government containment measures moderate depression by promoting trust and ease uncertainty (30), timely implementation of early screening and intervention for mental health at-risk individuals should be in place to prevent severe COVID-19 cases.****”****(Line 93-94*

g) Line 95. ''…this should not delay any public…'' What is '''this' referring to?

- ***Response: Kindly refer to the highlighted statement shown below:***

***"****Even though evidence showed stringent government containment measures moderate depression by promoting trust and ease uncertainty (30), timely implementation of early screening and intervention for mental health at-risk individuals should be in place to prevent severe COVID-19 cases.”(Line 94-95)*

3. Methods
a) Line 138. How the data on psychiatric illness was obtained?

- ***Response:* *Thank you for the question.***

***“*** *The history of psychiatric illness was a self-reporting response under the sub-heading of the presence of chronic illness (health domain) in the socio-demographic survey form.”(line 142-144)*

b) Line 139. The whole household where non-Malaysians lived was excluded?

- ***Response:* *Thank you for the question.***

*The enumerators have made sure no Malaysian was in the respective house before they exclude the whole household.*

c) Line 144. ''…Since this is a face-to-face…'' – change to was. Keep your past and present tense consistent throughout.

- ***Response:* Thank you for the reminder. The correction has been implemented:**

*“Since this was a face-to-face data collection, each enumerator was briefed about the University of Malaya COVID-19 Fieldwork Safety Protocol.”(line 129-130)*

4. Results
a) Line 247-248. Of 504 eligible participants, 432 (82.7%) completed the survey.

- ***Response***: Correction has been done. “Of 504 total eligible participants, 432 (82.7%) completed the survey.”(line 231)

b) Table 1.

I cannot find household income before Covid-19 in this table.

- ***Response: Apologies for the lack of information on the pre-Covid-19 income. Now we have added the information in the Table 1.***

Secondary schools and more than secondary schools % are in brackets. Is there a reason why this is different to other data presented in this table?

- ***Response*: *Thank you for the question. Our apologies, we have overlooked, and we have removed the brackets.***

Employed: yes/no or employment status: employed/not employed. 'Employment 'status' does not define if you are or are not employed.

- ***Response*: *The questionnaire is in Malay language, and participants were required to indicate whether if they are currently employed or not. Therefore, we have amended the table input as below:***

*“Are you currently employed or working (Before COVID-19)? “*

*“Are you currently employed or working (After COVID-19)?”*

Employment status (during Covid-19). Change the order of yes and no lines, so it matches the order of the employment status (before Covid-19).

- ***Response*: *Our apologies, and we have amended the input****.*

Household size. =4 belongs to which bracket?

Household size (person)

- ***Response: Our apologies and the shortcoming have been rectified:***

***Less than 4 (<4)***

***More than or same as 4 (≥4)***

House ownership. What is inherited mean? 'Aren't they home owners?

- ***Response: Thank you for your questions. Below is our explanation:***

*"Home owners" mean they are the first owner and may have still served the house loan, whereas inherited means it is property passed down from their ancestor, and they are part of the ownership sharing with their siblings. This reflects their socio-economic status".*

c) Lines 256-257. Did you mean 'Structural and socio-economic domains scores of poverty attribution were…'? It reads as if you are providing scores for three domains.

- ***Response: Thank you for the question. We have rephrased the statement:***

*"Poverty attribution of structural (3.6 (SD 0.8)) and socio-economic (3.8 (SD 0.9)) domains achieved the highest total mean scores out of the four domains of PA-21 (line 238-240)".*

d) Lines 262 – 264. Not clear. Is this 30% of male respondents or 30% of all participants? 130 is a third of your entire cohort, but this reads as if you are talking about male respondents only. n=278 male 130/278 (xxx%) used substances

- ***Response: Apology for the typo. The statement was rephrased:***

*“Among 130 (30.2%) respondents, substance use was present, with tobacco, alcohol, and sleeping pills being the most used substances. .”(line 247-248)*

e) Line 264-265. Is this sentence still describing male participants only?

- ***Response: Apology for the typo. The statement was rephrased:***

*“Substance use was present in 130 (30.2%) respondents, with tobacco, alcohol, and sleeping pills being the most used substances .”(line 245-246)*

f) Line 266. ''(n=30)'' Is this a typo?

- ***Response: Apology for the typo. The shortfall was rectified.***

g) Line 272-279. age less than 30... is a variable 'those aged less than '30' refers to participants. You talking about participants not variables here. This applies to first two sentences in this paragraph. Try to re-phrase if you want to write about factors.

- ***Response: Thank you for the responses. We have rephrased the statement:***

*“Factors positively associated with mild to severe symptoms of depression entail the age group of "less than 30 years" OR 5.11 (95%CI 2.04, 12.83), self-reported hypertension, having other chronic illnesses, and having the presence of past stressful life events (physical assault, long term illness, family issues and workplace issues)” (Line 254-257).*

h) Table 2. Sleep pill usage. 'Reference' duplication. Describe all the abbreviations used in this table.

- ***Response: Thank you for the reminder. Corrections have been done in Table 2.***

5. Discussion

a) Line 319-320. Suggest use 'respondents' here instead of group and delete word 'respondents' after 'financial strain'.

- ***Response: Thank you for the suggestion. We have edited the sentence.***

*"Lower-income respondents with higher financial strain were more likely to experience mild to severe depression (28).”(Line 298-299)*

b) Line 332. It would be good to see some numbers found in studies you're including in your reference index to support your statement.

- ***Response: Thank you for the suggestion. We have edited the sentence.***

*“The overall prevalence for poorer quality of life was 27.8%, comparable to a validation study from Trinidad and Tobago (28.0%) (71) but lower than the majority of the pre-pandemic studies from other countries (EQ-5D: 54.0%-69.7%) (72-75). The figure remain low even after accounting for socio-demographic and chronic illness characteristics (72,76).” (line 311-315)*

c) Line 338. Delete 'were'.

- ***Response: The changes have been done.***

d) Line 388.'...to the mid-20s to the 30s of age' – fix wording

- ***Response: Correction has been made: "…20 to 30-years of age…" at line 367***

e) Line 434. Cardiovascular problems? Adverse cardiovascular outcomes? Use something similar instead just 'cardiovascular outcomes'.

- ***Response: Thank you for the suggestion. We have made the changes:***

*"…vascular stiffening and endothelial dysfunction leading to adverse cardiovascular outcomes (117)." (Line 406)*

f) Line 466. Not sure what you mean by 'anxiety symptoms but not for depression'

- ***Response: Thank you for the response. Here is the improved version:***

*“The final analysis revealed, those with higher self-stigma scores were likely to have anxiety symptoms, whereas lower mental health-seeking attitudes predicted higher depressive* *symptoms. Poor help-seeking attitude prevents the low-income respondents from getting earlier treatment for severe mental health issues. The negative evaluations of professionals derived from negative past experiences and mistrust of the mental health professionals possibly deter a person from seeking help (125).” (Line 426-433)*

g) Line 469-470. Think about re-writing to avoid repetitions

- ***Response: Thank you for this response. Here is the improved version:***

*“The final analysis revealed that those with higher self-stigma scores were likely to have anxiety symptoms, whereas lower mental health-seeking attitudes predicted higher depressive symptoms. Poor help-seeking attitude prevents the low-income respondents from getting earlier treatment for severe mental health issues. The negative evaluations of professionals derived from negative past experiences and mistrust of the mental health professionals possibly deter a person from seeking help (125).”(Line 426-433)*

h) Line 477. Delete 'a’ in ‘…of health a promotion’

- ***Response: “a” was removed at line 439***

i) Line 494. Did you mean ‘…structural issues were more likely to experience…’

- ***Response:*** *Thank you for your response. The correction has been made at line 453*

j) Line 538-539. One third had low resilience and higher resilience scores. Isn't is mutually exclusive?

- ***Response: Apologies for the confusion. The sentence has now been rephrased to:***

*“Approximately one-third of respondents had low resilience. Higher resilience scores associated with lower depressive and anxiety symptoms.”(line 470-471)*

k) Line 539-540. ‘Substantial empirical studies have supported the inverse resilience scores concerning depression and anxiety’. Not sure what this sentence means.

- ***Response: Apologies for the confusion. The sentence now rephrased to:***

*“The findings were supported by a meta-analysis revealing of the association between inversed resilience scores with depression and anxiety (135).”(line 471-473)*

**Reviewer # xx**

1. Abstract:
a) Clearly written and concise.

- ***Response : Thank you***

2. Introduction
a) Presented relevantly.

- ***Response : Thank you***

3. Methods
a) Clearly presented.
b) However, regarding two of the tools used:

1.	EQ-5D

2.	SCSRFQ

As mentioned in the manuscript (line 134: The respondents completed multiple standardized instruments in the Malay language with assistance from the research team), were the tools mentioned above translated and validated in Malay language for this study? The other tools were clearly informed with reference regarding its validity of Malay language questionnaires.

- ***Response :***

***Both tools were validated in the Malay language but among different populations (1. Shafie et al & 2. Mohamad et al ). Therefore they were validated in the initial pilot test and results showed a Cronbach alpha score of more than 0.80.***

***The statement has been amended to :***

*“The Malay language of SCSRF has a Cronbach’s alpha of 0.84 and was validated in the current research’s pilot study with a good internal reliability of Cronbach’s alpha of 0.79.”(line 174-175)*

1. Shafie A, Vasan Thakumar A, Lim C, Luo N. Psychometric performance assessment of Malay and Malaysian English version of EQ-5D-5L in the Malaysian population. Qual Life Res. 2018.
2. Mohamad AS, Draman S, Aris MAM, Musa R, Rus RM, Malik M. Depression, anxiety, and stress among adolescents in Kuantan and its association with religiosity-a pilot study. IIUM Medical Journal Malaysia. 2018;17(2).
4. Statistical Analysis:
a) Not an expert to comment on this but the presentation of data was clear and easily understood.

- ***Response : Thank you***

5. Results:
a) No comment

- ***Response : Thank you***

6. Discussion:
a) It addresses the results adequately and well thought through. Findings are highlighted with relevant suggestions on the importance of mental health and psychosocial interventions that can be taken up to policymakers. Limitations were addressed.

- ***Response : Thank you***
